# Supplementary material for: GC-IMS-Based Volatile Characteristic Analysis of Hypsizygus marmoreus Dried by Different Methods
Source: Foods. 2024 Apr 25;13(9):1322. doi: 10.3390/foods13091322 (PMC11083298; doi:10.3390/foods13091322)
Supplement: Supplementary file 1 [file foods-13-01322-s001.zip › foods-2972609-supplementary.pdf]

Table S1 Gas chromatography program conditions.

Time (min)	Drift gas (mL/min)	Carrier gas (mL/min)	Recording
0	150	2	rec
2	150	2	-
10	150	10	-
20	150	100	-
40	150	100	stop

Table S2 GC-IMS analysis conditions.

**GC-IMS**	
Analysis time	40 min
chromatographic column	MXT-WAX (30 m× 0.53 mm× 1 μm)
Column temperature	60℃
Carrier gas/Drift gas	N_2
IMS temperature	45℃
**Automated headspace sampler**	
Injection volume	500 μL
Incubation time	20 min
Incubation temperature	60℃
Injection needle temperature	85℃
Incubation speed	500 rpm

Table S3 Total peak intensity of volatile compounds

Compound	HAD	HPD	HFD	UFD
Ester	57749.18±1645.53^{a}	58616.34±1823.31^{a}	37526.87±1142.76^{c}	42821.6±487.72^{b}
Aldehyde	43672.23±1224.98^{b}	45967.2±562.19^{a}	42330.17±255.35^{b}	34232.11±266.53^{c}
Alcohol	69547.58±1584.08^{a}	59759.48±398.29^{d}	64949.65±688.18^{c}	67253.74±339.13^{b}
Ketone	31337.91±1695.68^{a}	20903.29±81.83^{b}	21141.37±683.66^{b}	19118.7±406.56^{b}
Heterocyclic compound	8454.45±313.69^{a}	5443.76±82.71^{b}	5697.97±228.32^{b}	3987.23±37.23^{c}
Carboxylic acid	169684.85±3386.03^{a}	112958.72±5135.23^{b}	107790.6±3056.05^{b}	108074.25±964.34^{b}
Terpene	4852.6±122.64^{a}	4249.2±65.77^{b}	3362.57±70.83^{d}	3680.95±89.49^{c}
Sulfur-containing compound	1326.96±88.41^{d}	1845.21±15.2^{c}	3026.78±64.68^{a}	2100.46±19.72^{b}
Nitrogen-containing compound	16689.58±1745.14^{c}	22996.53±371.18^{b}	27988.61±2256.53^{a}	30365±1623.45^{a}
Aromatic hydrocarbon	760.66±23.35^{b}	719.13±15.16^{b}	850.45±52.77^{a}	419.44±14.21^{c}
Total	404076±1537.27^{a}	333458.86±3149.11^{b}	314665.04±5207.8^{c}	312053.48±366.87^{c}

The different letters within the same row indicate significant differences ($p < 0.05$).

Table S4 Percentage of volatile compounds

Compound	HAD (%)	HPD (%)	HFD (%)	UFD (%)
Ester	14.29±0.36	17.58±0.7	11.93±0.42	13.72±0.14
Aldehyde	10.81±0.26	13.78±0.05	13.46±0.17	10.97±0.1
Alcohol	17.21±0.33	17.92±0.29	20.64±0.23	21.55±0.09
Ketone	7.75±0.39	6.27±0.08	6.72±0.29	6.13±0.13
Heterocyclic compound	2.09±0.07	1.63±0.01	1.81±0.04	1.28±0.01
Carboxylic acid	42.00±0.98	33.86±1.22	34.25±0.47	34.63±0.27
Terpene	1.20±0.03	1.27±0.03	1.07±0.02	1.18±0.03
Sulfur-containing compound	0.33±0.02	0.55±0.01	0.96±0.03	0.67±0.01
Nitrogen-containing compound	4.13±0.45	6.9±0.17	8.89±0.61	9.73±0.53
Aromatic hydrocarbon	0.19±0.01	0.22±0.01	0.27±0.02	0.13±0.01
Total	100.00	100.00	100.00	100.00
